# Genetic characterisation of the rabies virus vaccine strains used for oral immunization of foxes in Poland to estimate the effectiveness of vaccination

**DOI:** 10.1007/s00705-014-2269-y

**Published:** 2014-11-19

**Authors:** Anna Orłowska, Jan Franciszek Żmudziński

**Affiliations:** Department of Virology, National Veterinary Research Institute, Partyzantów 57 Avenue, 24-100 Puławy, Poland

## Abstract

The main reservoir of rabies virus in Poland has been the red fox. To control rabies in wildlife, oral immunization of foxes was introduced in 1993. The vaccine is effective when it confers immunity against the virus circulating in the environment. To assess the above issue, a study of the molecular characteristics of 570-bp fragments of the N and G genes of vaccine strains SAD B19 and SAD Bern against street virus strains was performed. The results confirmed the similarity of the vaccine strains and rabies virus strains circulating in the environment and also demonstrate the genetic stability of vaccine strains that have been distributed in Poland for 20 years.

Rabies, a fatal zoonosis caused by rabies virus (RV), a member of the order *Mononegavirales*, family *Rhabdoviridae*, genus *Lyssavirus*, is distributed globally with the exception of some islands, archipelagos and countries. In Europe, wild animals, especially red foxes (*Vulpes vulpes*) [11, 15, 18, 22] and raccoon dogs (*Nyctereutes procyonoides*) [5, 23], are the main reservoir of rabies virus. Initially, the control of rabies outbreaks in wildlife was aimed at reduction of the fox population. That strategy has not prevented the spread of the disease. Oral rabies vaccination (ORV) of foxes is the only effective strategy for rabies eradication in wildlife [4, 5, 10, 12, 16, 17]. All of the commercially available oral vaccines, such as the modified live RV vaccines SAD B19, SAD Bern, SAG-1 and SAG-2, are derived from a common ancestor, the Street Alabama Dufferin (SAD) strain, which isolated from a naturally-infected dog in North America in 1935 [7, 13, 19]. Poland started oral vaccination of foxes in 1993 with Fuchsoral. A few years later, Lyssvulpen was introduced into the ORV program.

Rabies virus encodes five structural proteins: nucleoprotein (N), phosphoprotein (P), matrix protein (M), glycoprotein (G) and RNA-dependent RNA polymerase (L). The glycoprotein is the main antigen capable of inducing production of neutralizing antibodies (VNA)—a major immune effectors against infection [2, 6]. The G protein is also considered to play an important role in the pathogenesis of rabies virus by presenting a determinant located in antigenic site III.

The main objective of this study was to compare rabies virus strains contained in the vaccines Fuchsoral (SAD B19) and Lysvulpen (SAD Bern), which are used for ORV of foxes in Poland, to rabies virus strains collected from the field i.e. field (street) virus. The study was designed to provide information on the efficacy of the vaccine strains against the field viruses during the 20 year of rabies eradication. At the same time, the genetic stability of the vaccine strains was evaluated. Although the master seed virus used for vaccine production undergoes only a few passages in cell culture, making the risk of mutation is very small if not negligible, field RV strains undergo passage in animals of various species and therefore can escape from the protection induced by oral vaccines.

To investigate the genetic compatibility of vaccine and street RV strains, 50 vaccine samples (28 samples of SAD B19 and 22 samples of SAD Bern) originating from various batches of Fuchsoral and Lysvulpen and 44 Polish RV isolates collected in Poland between 1992 and 2014 were studied. Field samples were obtained from regional veterinary laboratories as brain samples that were positive for rabies virus by fluorescent antibody test (FAT) [8] with anti-nucleocapsid conjugate (Bio-Rad). As references, strains SAD B19 and SAD Tübingen, purchased from the WHO Reference Laboratory for Rabies (Pasteur Institute, Paris, France), were used. A detailed description of rabies virus strains included in the study is presented in Table 1.

**Table 1:** RV strains included in this study

RV strain designation	Vaccine name	Collection/vaccine batch	Collection date	GenBank accession number (N)
SADB19/1996/POL	Fuchsoral	1061	1996	KJ 513022
SADB19/1996/1/POL	Fuchsoral	12701	1996	KJ 513023
SADB19/1996/2/POL	Fuchsoral	10701s	1996	KJ 513024
SADB19/1996/3/POL	Fuchsoral	11801	1996	KJ 513025
SADB19/2000/POL	Fuchsoral	17501	1998	KJ 513026
SADB19/2000/1/POL	Fuchsoral	15801	1998	KJ 513027
SADB19/2000/2/POL	Fuchsoral	16901	1998	KJ 513028
SADB19/2000/3/POL	Fuchsoral	18901	1998	KJ 513029
SADB19/2004/POL	Fuchsoral	29801	2002	KJ 513030
SADB19/2004/1/POL	Fuchsoral	31001	2002	KJ 513031
SADB19/2004/2/POL	Fuchsoral	32301	2002	KJ 513032
SADB19/2004/3/POL	Fuchsoral	33601	2002	KJ 513033
SADB19/2008/POL	Fuchsoral	5120905	2006	KJ 513034
SADB19/2008/1/POL	Fuchsoral	5181005	2006	KJ 513035
SADB19/2008/2/POL	Fuchsoral	5470306	2006	KJ 513036
SADB19/2008/3/POL	Fuchsoral	5560406	2006	KJ 513037
SADB19/2010/POL	Fuchsoral	6970310	2010	KJ 513038
SADB19/2010/1/POL	Fuchsoral	6980310	2010	KJ 513039
SADB19/2010/2/POL	Fuchsoral	7160710	2010	KJ 513040
SADB19/2010/3/POL	Fuchsoral	7130710	2010	KJ 513041
SADB19/2012/POL	Fuchsoral	7751011	2012	–
SADB19/2012/1/POL	Fuchsoral	7800212	2012	–
SADB19/2012/2/POL	Fuchsoral	8040712	2012	–
SADB19/2012/3/POL	Fuchsoral	7920612	2012	–
SADB19/2014/POL	Fuchsoral	8880114	2014	–
SADB19/2014/1/POL	Fuchsoral	8710713	2014	–
SADB19/2014/2/POL	Fuchsoral	9040314	2014	–
SADB19/2014/3/POL	Fuchsoral	9050314	2014	–
SADBern/2003/POL	Lysvulpen	3711	2004	KJ 513042
SADBern/2003/1/POL	Lysvulpen	2811	2004	KJ 513043
SADBern/2003/2/POL	Lysvulpen	4511	2004	KJ 513044
SADBern/2003/3/POL	Lysvulpen	4211	2004	KJ 513045
SADBern/2006/POL	Lysvulpen	9613	2006	KJ 513046
SADBern/2006/1/POL	Lysvulpen	8513	2006	KJ 513047
SADBern/2006/2/POL	Lysvulpen	713	2006	KJ 513048
SADBern/2006/3/POL	Lysvulpen	1513	2006	KJ 513049
SADBern/2008/POL	Lysvulpen	3915	2008	KJ 513050
SADBern/2008/1/POL	Lysvulpen	3615	2008	KJ 513051
SADBern/2008/2/POL	Lysvulpen	3014	2008	KJ 513052
SADBern/2008/3/POL	Lysvulpen	3215	2008	KJ 513053
SADBern/2010/POL	Lysvulpen	9416	2010	KJ 513054
SADBern/2010/1/POL	Lysvulpen	9516	2010	KJ 513055
SADBern/2010/2/POL	Lysvulpen	1317	2010	KJ 513056
SADBern/2010/3/POL	Lysvulpen	1417	2010	KJ 513057
SADBern/2012/POL	Lysvulpen	8518	2012	–
SADBern/2012/1/POL	Lysvulpen	8819	2012	–
SADBern/2012/2/POL	Lysvulpen	1419	2012	–
SADBern/2012/3/POL	Lysvulpen	1119	2012	–
SADBern/2014/POL	Lysvulpen	9720	2014	–
SADBern/2014/1/POL	Lysvulpen	9320	2014	–
**Vaccine strains precursors**				
SAD B19 (SAD B19 Bern-C (Opava) passage from 1994)		CRBIP 8.19 (3805)	1995	
SAD Tübingen (Tubingen B-19 passage from 1994)		CRBIP 8.17 (2805)	1995	

Field RV strains	Host species	Collection date	GenBank accession number (N)	
A/1993/L/POL	Fox	1993	JN190357	
A/1994/L/POL	Fox	1994	JN190358	
C/1994/L/POL	Fox	1994	JN190359	
C/1994/L/1/POL	Fox	1994	–	
D/1994/B/POL	Cattle	1994	JN190361	
D/1992/L/POL	Fox	1992	JN190362	
R/1994/L/POL	Fox	1994	–	
R/1996/L/POL	Fox	1996	JN190364	
O/1994/L/POL	Fox	1994	JN190365	
F/1994/L/POL	Fox	1994	–	
L/1994/L/POL	Fox	1994	JN190367	
K/1994/K/POL	Cat	1994	–	
B/1995/L/POL	Fox	1995	JN190369	
H/1996/L/POL	Fox	1996	–	
P/1996/P/POL	Dog	1996	JN190371	
M/1996/L/POL	Fox	1996	JN190372	
L/2000/L/POL	Fox	2000	JN190373	
D/2001/L/POL	Fox	2001	JN190374	
C/2002/L/POL	Fox	2002	JN190375	
F/2001/L/POL	Fox	2001	JN190376	
H/2000/L/POL	Fox	2000	JN190377	
P/2000/L/POL	Fox	2000	JN190378	
B/2003/L/POL	Fox	2003	JN190379	
O/1999/L/POL	Fox	1999	–	
M/2001/L/1/POL	Fox	2001	–	
N/2001/L/POL	Fox	2001	JN190382	
J/2003/L/POL	Fox	2003	JN190383	
E/1992/K/POL	Cat	1992	JN190385	
E/2003/L/POL	Fox	2003	JN190386	
E/2004/L/POL	Fox	2004	–	
E/2003/K/POL	Cat	2003	JN190387	
E/2007/L/POL	Fox	2007	JN190389	
J/2008/L/POL	Fox	2008	–	
H/2008/B/POL	Cattle	2008	JN190391	
H/2008/J/POL	Raccoon dog	2008	JN190392	
O/2009/L/POL	Fox	2009	JN190393	
N/2008/K/POL	Cat	2008	–	
P/2010/L/POL	Fox	2010	JN190395	
N/2010/L/POL	Fox	2010	–	
O/2012/L/POL	Fox	2012	–	
P/2012/L/POL	Fox	2012	–	
M/2014/L/POL	Fox	2014	–	
P/2014/L/POL	Fox	2014	–	
N/2014/L/POL	Fox	2014	–	

Total RNA was extracted using a commercial kit QIAamp Viral RNA Mini Kit (QIAGEN) according to the manufacturer’s instructions. To amplify a 600-bp fragment of the RV nucleoprotein gene, a previously published method was used [20]. RT-PCR was carried out using the primers JW12 and JW6DPL, which were described by Heaton et al. [14]. A 590-bp fragment of the G gene corresponding to nt 3957–4547 of the PV reference strain (accession no. M13215), encoding antigenic site III of the RV glycoprotein, was amplified as described in a previous paper (DOI 10.1007/S00705/014-2045-z). Following amplification, PCR products were sequenced using an automated sequencer (ABI PRISM 310 Genetic Analyzer, Applied Biosystems) with a BigDye Sequencing Kit (Applied Biosystems) and GeneScan Analysis Software. The nucleotide sequences were assembled using CAP3 software. Multiple sequence alignments were done based on the 570-bp regions of the nucleoprotein and glycoprotein genes. To assess the genetic compatibility of vaccine and the street RV strains, the nucleotide sequences were translated into amino acid sequences and aligned using BioEdit software v. 7.0.5.3. Phylogenetic trees were generated using MEGA software v. 5.0 [21] using the neighbour-joining (NJ) method with the Kimura 2-parameter model. Bootstrap values were calculated for a set of 1000 replicates. Reference sequences of SAD B19 (accession no. EF206709.1) and SAD Bern (accession no. EF206708.1) were used for phylogenetic analysis.

The pairwise comparison of 570-nt-long N and G gene fragments of vaccine strains SAD Bern and SAD B19 to reference strains delivered from the Pasteur Institute showed 100 % homology with the SAD B19 (CRBIP 8.17) and SAD Tübingen (CRBIP 8.19) strains, with the exception of the SAD Bern/2006/2/POL Lysvulpen strain (batch no. 713), which was distributed in Poland in 2006 and had a single substitution of A to G at position 200 in the analyzed N gene fragment. Additionally, all of the vaccine samples analyzed (SAD B19 and SAD Bern), irrespective of their production date, had 99.8–100 % homology in the analyzed 570-nt fragment of the N gene and 100 % homology within the analyzed nucleotide sequences of the G gene. Moreover, no differences in the nucleotide sequences of the 570-long fragments of the N and G genes were found between SAD Bern strains collected from the Lysvulpen vaccine and SAD B19 strains collected from the Fuchsoral vaccine. The SAD B19 and SAD Bern samples were almost 100 % identical in sequence with the exception of the single nucleotide substitution in the above-mentioned SAD Bern/2006/2/POL strain. This substitution was nonsynonymous, and thus the amino acid sequence identity was 99.8–100 %. Also, phylogenetic analysis of the dataset from nucleotide sequences of vaccine strains against reference sequences from GenBank and the strains from the Pasteur Institute showed that all of the RV strains extracted from Fuchsoral and Lysvulpen formed one group of SAD vaccine virus strains based on the analysed fragments of the N and G genes (Fig. 1).

**Fig. 1:**
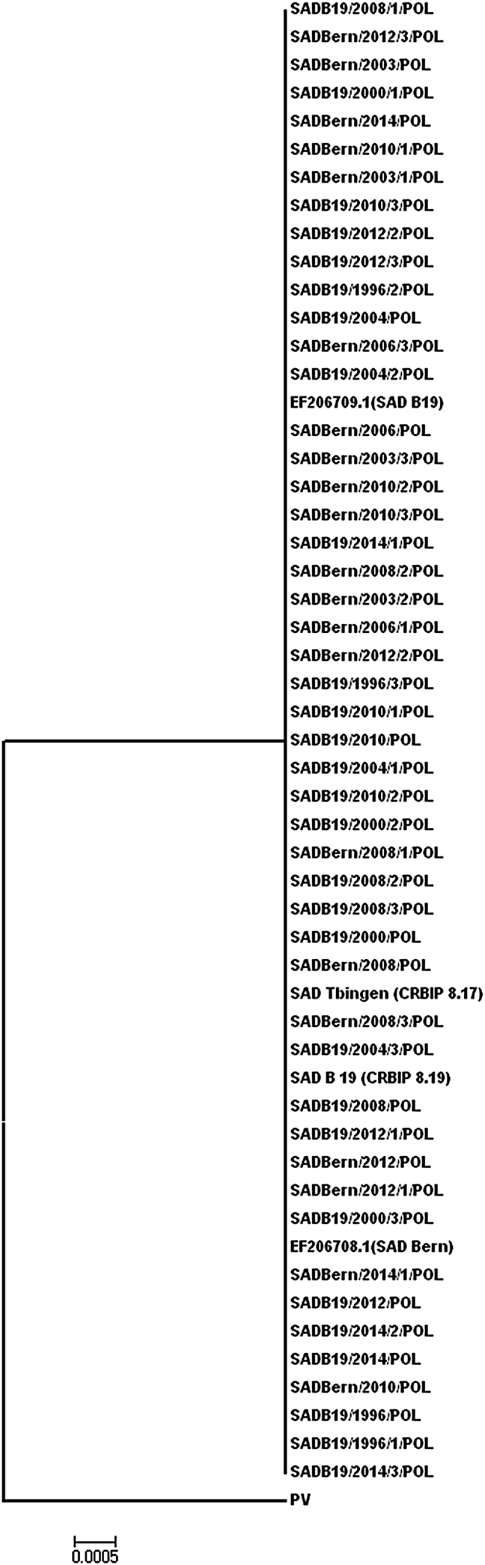
Phylogenetic tree comparing the SAD B19 and SAD Bern strains collected from Fuchsoral and Lysvulpen vaccines with reference strains. The phylogenetic analysis was based on fragments of N and G gene nucleotide sequences, using the neighbor-joining method

Phylogenetic analysis of amino acid sequences within antigenic site III of Polish field RV strains collected between 1992 and 2014 and the vaccine strains isolated from Lysvulpen and Fuchsoral distributed in Poland between 1996 and 2014 showed a very high homology, with the exception of a single substitution of isoleucine for valine at position 136 in the amino acid sequence of the M/2001/L1/POL strain. Figure 2 shows an alignment of the amino acid sequences within antigenic site III. Based on the analysed fragments of the RV genome and the corresponding aa sequences, the results confirm a very high compatibility of vaccine strains with the field strains.

**Fig. 2:**
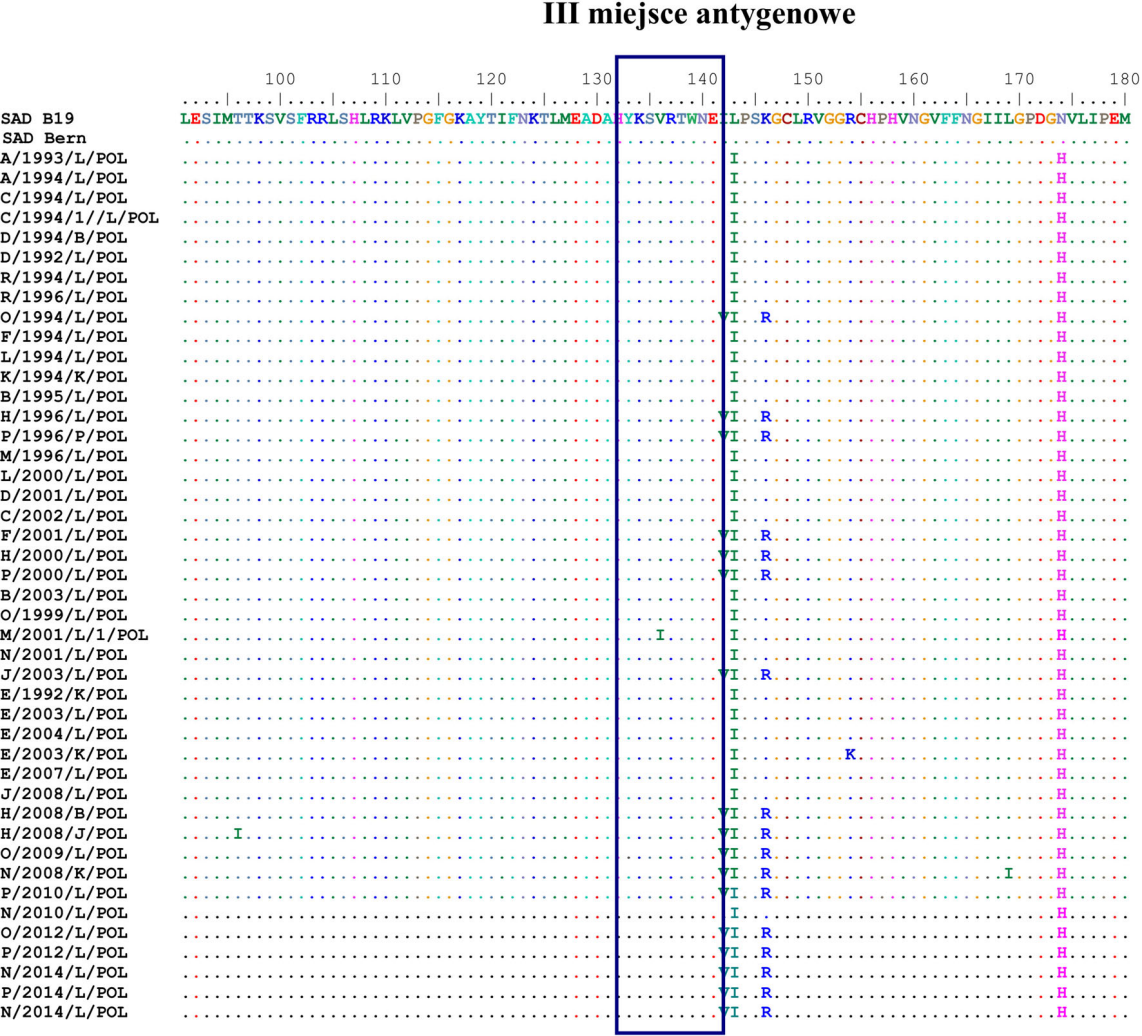
Alignment of deduced amino acid sequences of glycoprotein fragments of SAD B19 and SAD Bern vaccine strains as well as rabies virus field strains. The box indicates the amino acid sequence of antigenic site III of the rabies virus glycoprotein

The issue of the compatibility of the RV strains applied in ORV and RV field isolates has become even more important in relation to an outbreak of rabies that occurred in Poland, in Malopolska voivodeship, in 2010. The region had been free of rabies for 7 consecutive years. The analysis of the effect of ORV in Malopolska based on antibody response (86 % seropositive reactors) and the rate of bait uptake (tetracycline in fox bones, approx. 80 %) could suggest that the rabies virus escape mutant appeared in the field and that the vaccine does not protect animals against the onset of rabies. From 20 August to 31 December 2010, 118 rabies cases were diagnosed, mostly affecting foxes in a region that was temporarily free of rabies. The study has confirmed the similarity of the attenuated vaccine strains with rabies virus circulating in the field based on the aa sequences of a fragment of the G protein that is crucial for immunity against rabies. A detailed phylogenetic analysis of a short fragment (570 bp) of the nucleoprotein gene of field rabies isolates and the SAD Bern strain used for oral vaccination in Malopolska voivodeship has excluded an involvement of the vaccine strain in the rabies outbreak and has shown a close relationship of Polish RV field strains to Ukrainian and Romanian RV strains (NEE group). Further PCR-RFLP analysis based on an N gene fragment confirmed that the Polish RV strains were field strains (data not presented). Thus, the appearance of rabies epidemics in Malopolska voivodeship appears to have resulted from migration of rabid animals from outside of the region or smuggling (illegal introduction) of rabid animals rather than reversion of attenuated oral vaccines against rabies to a pathogenic form. Detailed analysis of rabies epidemiology has revealed that the rabies occurs in urban foxes, a population that is beyond the reach of vaccines distributed by plane. Thus, the responsible authority has decided to distribute the vaccine by hand in urban areas. A preliminary evaluation of the action that was taken has had indicated an improvement of the epidemic situation regarding rabies in Malopolska voivodeship.

Amino acid sequence the comparisons were carried out for antigenic site III of the ectodomain of the rabies virus glycoprotein. It is known that the ectodomain of rabies the virus glycoprotein carries five antigenic sites and that sites II and III are essential for pathogenesis and the stimulation of production of rabies-virus-neutralizing antibodies [9]. Despite the fact that oral vaccines are intended for foxes, the present study also involved RV strains isolated from domestic animals because field strains can infect wild and domestic animals. The study demonstrated almost 100 % homology between vaccine strains and the Polish RV fields strains within aa sequences of antigenic site III. A single substitution of V by I in the Polish M/2001/L1/POL strain was the only mutation observed in the 44 isolates that were analyzed. This suggests that the SAD B19 and SAD Bern vaccine strains are effective in rabies eradication and that they should confer protection against the infection with field viruses. Therefore, the hypothesis that RV field strains are subjected to immune pressure from the immune population, resulting in the appearance of RV escape mutants that are not compatible with the vaccine strains SAD B19 and SAD Bern, is not supported by the data.

A comparison of the nucleotide and amino acid sequences of vaccine strains collected from the oral vaccines Fuchsoral and Lysvulpen showed 100 % nucleotide sequence identity and 100 % homology to the reference strains SAD Tübingen (8.17) and SAD B19 (8.19) obtained from the Pasteur Institute. Although Cliquet et al. [3] have pointed out the possibility of genetic modification of a live rabies virus vaccine strain (marketed as the SAD Bern strain) against reference vaccine strains, our results based on two fragments of the N and G genes suggest that the vaccine strains contained in the commercial vaccines retain the characteristics of the master seed strains during serial passages within the production cycle. Homology at the level of 99.8–100 % was found between the SAD Bern and SAD B19 strains within 570-nt fragments of the N and G genes. This confirms the previously reported high conservation and genetic stability of SAD B19 strains passaged several times in mice [1]. It can be concluded that the virus strains used for production of oral vaccines, Fuchsoral and Lysvulpen, did not change during the years 1996–2014. The only change that was observed within the N gene fragment in strain SAD Bern/2006/POL was a single substitution of G for A at position 198. The appearance of a non-synonymous mutation was surprising. Unfortunately, information on the number of passages of this particular batch of vaccine is not available. However, according to the manufacturer’s declaration, the virus used in the oral vaccine must not be passaged more than five times. Thus, after careful consideration, we suppose that the appearance of the mutation was rather random.

The genetic identity of the SAD Bern and SAD B19 strains is obviously due to the fact that they were derived from a common ancestral SAD strain. The hypothesis has been presented that SAD B19 and SAD Bern strains are the same strain. Geue et al. [13] have shown close genetic relationships (99.346–99.991 % homology) of five RV vaccine strains collected from commercial vaccines distributed in Europe and a common origin from the same strain when compared to the original archived vaccine strains. In the present study, both of the analyzed fragments of the N and G genes showed a high degree of conservation. Therefore, no differences within nucleotide sequences between SAD Bern and SAD B19 strains were observed.

Rabies vaccines based on attenuated SAD strains are the most frequently used in Europe for oral vaccination due to their safety and good immunogenic properties, and for economics reasons. This study demonstrates the molecular compatibility of the vaccine strains with street RV strains, confirming that SAD B19 and SAD Bern are still effective in protection against field RV strains. The results demonstrate the genetic stability of the strains used for the production of a consecutive series of vaccines between 1996 and 2014 and also confirm that the vaccine is still potent against current field viruses. Monitoring of the effect of ORV in Poland has shown that, e.g., in 2013, around 77 % of foxes submitted for testing were seropositive, and about 84 % of foxes contained tetracycline in their bones. Also, epidemiological data showed a decrease in the number of rabies cases, from 3084 in 1992 (before the start of ORV in Poland) to 8 in 2009, confirming the efficacy and stability of the vaccine strains. Thus, other factors, such as bait integrity or the system of vaccine distribution (aerial distribution not reaching the urban foxes) have failed, playing an important role in rabies outbreaks in southern Poland in 2010–2013. As of September 2014, Poland has recorded a low number of rabies cases compared to the same period in 2012 and 2013.

## References

[1] Beckert A, Geue L, Vos A, Neubert A, Freuling C, Müller T (2009). Genetic stability (in vitro) of the attenuated oral rabies virus vaccine SAD B19. Microbiol Immunol.

[2] Benmansour A, Leblois H, Coulon P, Tuffereau C, Gaudin Y, Flamand A, Lafay F (1991). Antigenicity of rabies virus glycoprotein. J Virol.

[3] Cliquet F, Robardet E, Picard Meyer E (2013). Genetic strain modification of a live rabies virus vaccine widely used in Europe for wildlife oral vaccination. Antiviral Res.

[4] Cliquet F, Aubert M (2004). Elimination of terrestrial rabies in Western European countries. Dev Biol (Basel).

[5] Cliquet F, Robardet E, Must K, Laine M, Peik K, Picard Meyer E, Guiot AL, Niin E (2012). Eliminating rabies in Estonia. PLOS Negl Trop Dis.

[6] Cox JH, Dietzchold B, Schneider LG (1977). Rabies virus glycoprotein. II. Biological and serological characterization. Infect Immun.

[7] Conzelmann KK, Cox JH, Schneider LG, Thiel HJ (1990). Molecular cloning and 
complete nucleotide sequence of the attenuated rabies virus SAD B 19. Virology.

[8] Dean DJ, Abelseth MK, Atanasiu P, Meslin FX, Kaplan MM, Koprowski H (1996). The fluorescent antibody test. Laboratory techniques in rabies.

[9] Dietzschold B, Wunner BH, Wiktor TJ, Lopes AD, Lafon M, Shmitz CL, Koprrowski H (1983). Characterization of an antigenic determinant of the glycoprotein that correlates with pathogenicity of rabies virus. Proc Natl Acad Sci USA.

[10] Faber M, Dietzschold B, Li J (2009). Immunogenicity and safety of recombinant rabies viruses used for oral vaccination of stray dogs and wildlife. Zoonoses Public Health.

[11] Finnegan CJ, Brookes SM, Johnson N, Smith J, Mansfield KL, Keene VL, McElhinney LM, Fooks AR (2002). Rabies in North America and Europe. J R Soc Med.

[12] Freuling CM, Hampson K, Selhorst T, Schröder R, Meslin FX, Mettenleiter TC, Müller T (2013). The elimination of fox rabies from Europe: determinants of success and lessons fort he future. Phil Trans R Soc B.

[13] Geue L, Schares S, Schnick C, Kliemt J, Beckert A, Freuling C, Conraths FJ, Hoffmann B, Zanoni R, Marston D, McElhinney L, Johnson N, Fooks AR, Tordo N, Müller T (2008). Genetic characterisation of attenuated SAD rabies virus strains used for oral vaccination of wildlife. Vaccine.

[14] Heaton PR, Johnstone P, McElhinney LM, Cowley R, O’Sullivan E, Whitby JE (1997). Heminested PCR assay for detection of six genotypes of rabies and rabies-related viruses. J Clin Microbiol.

[15] Johnson N, Freuling C, Vos A, Un H, Valtchovski R, Turcitu M, Dumistrescu F, Vuta V, Velic R, Sandrac V, Aylan O, Müller T, Fooks AR (2008). Epidemiology of rabies in Southeast Europe. Dev Biol (Basel).

[16] Matouch O, Vitasek J, Semerad Z, Malena M (2007). Rabies—free status of the Czech Republic after 15 years of oral vaccination. Rev Sci Tech.

[17] Matouch O, Vitasek J, Semerad Z, Malena M (2006). Elimination of rabies in the Czech Republic. Dev Biol (Basel).

[18] Pötzsch CJ, Kliemt A, Klöss D, Schröder R, Müller W (2006). Rabies in Europe—trends and developments. Dev Biol (Basel).

[19] Sacramento D, Bourhy H, Tordo N (1991). PCR technique as an alternative method for diagnosis and molecular epidemiology of rabies virus. Mol Cell Probes.

[20] Smreczak M, Orlowska A, Trebas P, Zmudzinski JF (2008). Application of heminested RT-PCR to the detection of EBLV1 and classical rabies virus infections in bats and terrestrial animals. Bull Vet Inst Pulawy.

[21] Tamura K, Dudley J, Nei M, Kumar S (2007). MEGA4: molecular evolutionary genetics analysis (MEGA) software version 4.0. Mol Biol Evol.

[22] Wandeler AI (2008). The rabies situation in Western Europe. Dev Biol (Basel).

[23] Zienius D, Zilinskas H, Sajute K, Stankevicius A (2009). Comparative molecular characterization of the rabies virus in the Lithuanian raccoon dog population. Bull Vet Pulawy.

